# Ofatumumab in Myelin Oligodendrocyte Glycoprotein Antibody–Associated Disease: A Comparison With Rituximab

**DOI:** 10.1002/acn3.70392

**Published:** 2026-04-26

**Authors:** Yuxin Fan, Lei Zhou, Jingzi ZhangBao, Hongmei Tan, Zhouzhou Wang, Chao Quan

**Affiliations:** ^1^ Department of Neurology, Huashan Hospital, Shanghai Medical College, Fudan University, 12 Wulumuqi Road (Mid) Shanghai China; ^2^ National Center for Neurological Disorders Shanghai China

**Keywords:** B cell, monoclonal antibody, myelin oligodendrocyte glycoprotein antibody associated disease

## Abstract

**Objective:**

To evaluate the efficacy and safety of ofatumumab in patients with myelin oligodendrocyte glycoprotein antibody–associated disease (MOGAD), and compare it with rituximab.

**Methods:**

We conducted a single–center, observational study including 22 MOGAD patients treated with ofatumumab and 21 treated with rituximab. The primary outcome is relapse, while the secondary outcomes are disability status and adverse events.

**Results:**

Twenty–two patients received ofatumumab for a median duration of 19.5 months (range 6–41). Among them, 18 patients (81.82%) remained relapse–free during follow–up. Annualized relapse rate (ARR) significantly decreased from 1.30 (95% CI 0.74–2.29) pretreatment to 0.12 (95% CI 0.04–0.35) during ofatumumab therapy (IRR 10.86, 95% CI 3.22–36.70, *p* < 0.001). Through propensity score matching, 11 patients in the ofatumumab group and 11 patients in the rituximab group were compared. Ofatumumab was observed to be associated with a significantly lower risk of a second attack (HR 0.233, 95% CI 0.061 to 0.893, *p* = 0.018) compared to rituximab. Adverse events were reported in 54.55% patients (6/11) treated with ofatumumab, most of which were mild and occurred after the initial dose.

**Interpretation:**

Ofatumumab was associated with a lower relapse risk compared with rituximab in patients with MOGAD.

## 1 Introduction

Myelin oligodendrocyte glycoprotein antibody–associated disease (MOGAD) is an inflammatory demyelinating disorder of the central nervous system (CNS). The clinical spectrum of MOGAD includes acute disseminated encephalomyelitis (ADEM), optic neuritis (ON), myelitis, seizures, and encephalitis [1, 2, 3]. Although initially considered a less relapsing and relatively benign condition compared to neuromyelitis optica spectrum disorder (NMOSD) with aquaporin–4 (AQP4) antibodies, accumulating evidence from longitudinal studies has revealed a higher frequency of relapses and a greater risk of severe neurological disability than previously recognized [4, 5].

There is a growing need for effective management of MOGAD, while no approved treatment for MOGAD exists at present. Current therapeutic strategies are primarily based on uncontrolled studies, which have shown that azathioprine (AZA), mycophenolate mofetil (MMF), intermittent intravenous immunoglobulin (IVIg), rituximab (RTX), and tocilizumab are effective in reducing relapses of MOGAD [6, 7, 8, 9, 10, 11, 12, 13, 14, 15, 16, 17].

Rituximab (RTX) shows medium–term efficacy in MOGAD, albeit less pronounced than in AQP4 + NMOSD. Approximately 30%–50% of MOGAD patients experience relapses despite the biological effects of RTX [14, 15]. It has been proposed that certain MOGAD patients may possess an increased susceptibility to even minimal reappearance of circulating memory B cells, as disease relapses have been documented when their frequency approaches approximately 0.05% [14]. This finding diverges sharply from patterns observed in AQP4 + NMOSD, wherein most relapses manifest only when memory B cell levels surpass the threshold by a considerable margin [14]. Furthermore, pathogenic B cells residing in lymphoid tissues may contribute to relapse in MOGAD, but RTX is less effective at depleting these cells in that compartment [14, 15, 16, 17, 18, 19, 20]. Lastly, there is a possibility that the pathogenesis of MOGAD involves B cells with low CD20 expression, which RTX may not efficiently target [21].

However, this situation does not imply that all B cell–depleting agents are less effective in MOGAD. Ofatumumab, a fully human anti–CD20 monoclonal antibody, targets the discontinuous sequences of the small and large extracellular loops of CD20 [22]. It has a stronger binding affinity to the cell membrane and has a slower dissociation rate from CD20 than does rituximab [22, 23, 24, 25]. This stable binding not only supports antibody–dependent cellular cytotoxicity (ADCC) but also induces potent complement–dependent cytotoxicity (CDC)—enabling efficient lysis even of CD20–low B–cell subsets [24, 25, 26, 27]. The low–dose, monthly regimen provides sustained B–cell depletion, minimizing the risk of B cell repletion between doses [28, 29, 30]. Subcutaneous administration permits more direct lymphatic access compared with intravenous infusion, thereby targeting lymph node‐resident B cells [18, 19, 20].

Emerging evidence from recent case studies supports the therapeutic potential of ofatumumab in MOGAD [31, 32]. In this study, we investigated the efficacy and safety of ofatumumab in MOGAD, comparing it with RTX. We aim to explore its potential role as a novel second–line immunotherapy for MOGAD.

## 2 Methods

### 2.1 Study Population and Data Collection

From February 2016 to September 2025, 48 consecutive patients diagnosed with MOGAD were treated with either rituximab (RTX) or ofatumumab (OFA) at the Department of Neurology, Huashan Hospital. The first patient in this cohort received RTX in March 2020, while OFA was first administered in January 2022. Among them, 22 MOGAD patients treated with ofatumumab and 21 treated with rituximab were included in this study. The inclusion criteria were: (1) Diagnosis of MOGAD based on the 2023 diagnostic criteria [33]; (2) Treatment with ofatumumab or rituximab for at least 6 months; (3) No concomitant use of other immunosuppressants (including AZA, MMF, or tacrolimus) or other monoclonal antibody therapies. Intravenous immunoglobulin (IVIg) was not classified as an immunosuppressant. (4) Availability of clear clinical and therapeutic data in the hospital–based MOGAD database.

At Huashan hospital, patients with MOGAD were registered upon diagnosis. Baseline information, including disease history, clinical and paraclinical assessments, was collected at the time of registration. This included age of onset, sex, onset attack type, subsequent attack types, MOG–IgG titer, concomitant autoimmune disease, previous treatments, Expanded Disability Status Scale (EDSS) score, CSF parameters (cell count, protein, oligoclonal bands and IgG index), visual acuity, and magnetic resonance imaging (MRI) findings. Follow–up data were then recorded prospectively in the Database. Data of the patients treated with OFA or RTX were retrieved from this database in September 2025. Statistical analyses were conducted from September to October 2025.

### 2.2 Treatment Administration

Before maintenance treatment initiation, physicians provided patients with comprehensive information regarding available therapeutic options for MOGAD (oral immunosuppressants, tocilizumab, IVIg, RTX, and ofatumumab). Mechanisms of action, expected efficacy, potential adverse effects, cost, administration route and frequency were explained to the patients. Ultimately, treatment options were made through a process of shared decision–making between the patient and physician, incorporating considerations of the patient's individual clinical status and financial circumstances.

Ofatumumab was administered subcutaneously at 20 mg on Days 1, 7, and 14, followed by 20 mg every 4 weeks starting from Day 28. The RTX regimen applied is widely used in China, which consisted of 500 mg intravenously on Day 1 and another 500 mg on Day 2, with a repeated dose of 500 mg at fixed 6–month intervals thereafter. All patients receiving either ofatumumab or rituximab underwent screening for active hepatitis B and tuberculosis before treatment initiation.

The median body weight of pediatric patients (< 18 years old) treated with ofatumumab was 42.5 kg (range, 31–55 kg), whereas the median body weight of those treated with rituximab was 57.3 kg (range, 38–92 kg). In pediatric patients, those weighing ≥ 40 kg received ofatumumab according to the adult dosing regimen. Patients weighing < 40 kg were treated with 20 mg every 6 weeks following the loading dose. The rituximab regimen was adjusted according to body weight and body surface area. Pediatric patients weighing ≥ 50 kg were treated according to the adult regimen. Those weighing < 50 kg received rituximab intravenously at a dose of 375 mg/m^2^ on Day 1 and a second 375 mg/m^2^ dose on Day 2, followed by maintenance dosing of 375 mg/m^2^ every 6 months.

Notably, in our cohort, only three pediatric patients in the ofatumumab group weighed < 40 kg and therefore received the weight–adjusted regimen. Similarly, only two pediatric patients in the rituximab group weighed < 50 kg and were treated according to the body surface area–based regimen.

### 2.3 Definitions

Attacks/relapses were adjudicated by a neurologist who was blinded to treatment allocation and were defined as the worsening of existing symptoms or the occurrence of new symptoms lasting for at least 24 h without other aetiologies; multiple symptoms within 30 days were considered a single attack. Annualized relapse rates (ARRs) were determined by dividing the total number of relapses observed during the follow–up period by the total number of patient–years of follow–up. Disability was evaluated using Expanded Disability Status Scale (EDSS).

Patients with ≥ 1 clinical relapses were defined as having a relapsing disease course.

### 2.4 Outcomes

The primary outcome of the study is relapse, as measured by ARR and time to relapse, while the secondary outcomes are disability status and adverse events.

### 2.5 MOG‐IgG Detection

MOG–IgG was tested using live cell–based indirect immunofluorescence test (IIFT). HEK293 cells transfected with full–length human MOG were employed. A secondary antibody goat anti–human IgG Fcγ fragment–specific was used.

### 2.6 Statistical Analysis

Statistical analysis was performed by using SPSS (version 27.0) and R software (version 4.2), with GraphPad Prism (version 9.5.0) for graph creation. Categorical variables were presented as numbers (percentages) and were compared using the χ2 test or Fisher's exact test, as appropriate. Continuous variables are presented as medians with ranges, and they were compared using Student's *t–*test, Wilcoxon test or one–way analysis of variance. A *p* ≤ 0.05 was considered statistically significant.

Patients were matched in a 1:1 ratio using nearest–neighbor matching, applying a caliper width of 0.2 of the standard deviation of the logit of the propensity score. Covariate balance before and after matching was assessed using standardized mean differences (SMDs). The distribution of propensity scores in each treatment group before and after matching was visually inspected using density plots (Figure S3).

ARRs were analyzed using a negative binomial regression model. Follow–up time was incorporated as an offset using the natural logarithm of treatment exposure time. Treatment effects were assessed by including treatment group as a covariate in the model, and results are reported as incidence rate ratios (IRRs) with corresponding 95% CIs.

The Kaplan–Meier method with 95% CIs was used to compare the relapse–free duration of ofatumumab and rituximab. Survival curve comparison was conducted using the log–rank test, and data were used after PSM. Multivariate logistic regression analyses were performed to identify factors associated with a relapsing course after ofatumumab or rituximab initiation. *p* values of both univariate and multivariate analysis were adjusted using the Benjamini–Hochberg method.

### 2.7 Standard Protocol Approvals, Registrations, and Patient Consent

The study was approved by the medical ethics committees of Huashan Hospital, and written informed consent was obtained from each patient.

## 3 Result

### 3.1 Study Population

We included 22 MOGAD patients treated with ofatumumab and 21 treated with rituximab (Table 1). In the ofatumumab group, the median age at disease onset was 23.5 years (range, 4–56), while in the RTX group it was 20 years (range, 5–53) (*p* = 0.932). The median age at treatment initiation was 26.5 years (range, 7–58) for the ofatumumab group and 27 years (range, 10–54) for the RTX group (*p* = 0.903). Median disease duration before treatment was 25 months (range, 1–142) in the ofatumumab group and 14 months (range, 0.5–236) in the RTX group (*p* = 0.593). The median treatment duration for ofatumumab was 19.5 months (range, 6–41) in the ofatumumab group and 18 months (range, 6–44) in the rituximab group (*p* = 0.836).

**TABLE 1:** Baseline characteristics of patients treated with rituximab and ofatumumab, before and after propensity score matching.

Variable	Unadjusted			Matched		
	Rituximab (*n* = 21)	Ofatumumab (*n* = 22)	SMD	Rituximab (*n* = 11)	Ofatumumab (*n* = 11)	SMD
Age, *n* (%)						
Children	9 (42.86)	9 (40.91)	−0.040	4 (36.36)	3 (27.27)	−0.204
Adult	12 (57.14)	13 (59.09)	0.040	7 (63.64)	8 (72.73)	0.204
Gender, *n* (%)						
Female	9 (42.86)	16 (72.73)	0.671	8 (72.73)	8 (72.73)	0.000
Male	12 (57.14)	6 (27.27)	−0.671	3 (27.27)	3 (27.27)	0.000
Number of relapses before mAb, *n* (%)						
0	6 (28.57)	3 (13.64)	−0.435	1 (9.09)	2 (18.18)	0.236
1–3	7 (33.33)	9 (40.91)	0.154	5 (45.45)	5 (45.45)	0.000
≥ 3	8 (38.10)	10 (45.45)	0.148	5 (45.45)	4 (36.36)	−0.189
Onset phenotype, *n* (%)						
ON	7 (33.33)	7 (31.82)	−0.033	5 (45.45)	4 (36.36)	−0.189
Myelitis	3 (14.29)	1 (4.55)	−0.468	1 (9.09)	1 (9.09)	0.000
Brain	6 (28.57)	7 (31.82)	0.070	2 (18.18)	2 (18.18)	0.000
Mix	5 (23.81)	7 (31.82)	0.172	3 (27.27)	4 (36.36)	0.189
Maintenance therapy before mAb^a^, *n* (%)						
None	13 (61.90)	9 (40.91)	−0.427	5 (45.45)	6 (54.55)	0.183
At least 1 maintenance therapy	8 (38.10)	13 (59.09)	0.427	6 (54.55)	5 (45.45)	−0.183
Disease duration before treatment^b^, *n* (%)						
< 12 months	9 (42.86)	8 (36.36)	−0.135	4 (36.36)	5 (45.45)	0.183
≥ 12 months	12 (57.14)	14 (63.64)	0.135	7 (63.64)	6 (54.55)	−0.183

Abbreviations: mAb, monoclonal antibody; SMD, standardized mean difference.

^a^ Maintenance therapy before mAb refers to azathioprine (AZA), mycophenolate mofetil (MMF), tacrolimus, and tocilizumab.

^b^ Disease duration before treatment is the time span from the onset attack to ofatumumab or rituximab initiation.

Before ofatumumab, 13 patients (59.09%) had received prior maintenance therapies, including 12 with MMF, 2 with tacrolimus, and one each with IVIg and tocilizumab. Among RTX–treated patients, 8 (38.10%) had received maintenance therapy before, including 6 with MMF and one each with AZA and tacrolimus. During ofatumumab treatment, one patient received concomitant IVIg, and all patients received methylprednisolone at treatment initiation. In the rituximab group, no patients received IVIg, and one patient did not receive methylprednisolone.

Two patients began ofatumumab therapy after their first demyelinating episode. One was a woman in her late twenties who experienced her first attack 1.5 months postpartum. MRI revealed longitudinally extensive lesions in the medulla, cervical, and thoracic spinal cord, with an ‘H–sign’ appearance. Her peak EDSS score was 3. Despite high–dose steroid pulse therapy, she had persistent urinary and bowel dysfunction, resulting in a residual EDSS score of 2. The second patient, a man in his early forties, presented with unilateral optic neuritis, with the lowest visual acuity of hand motion. His visual acuity improved to 0.4 following corticosteroid pulse therapy. Given the significant neurological sequelae, and after a shared decision–making process between the patient and physician, both patients initiated ofatumumab treatment.

### 3.2 Comparison of Relapse and ARR With and Without PSM

**FIGURE 1:**
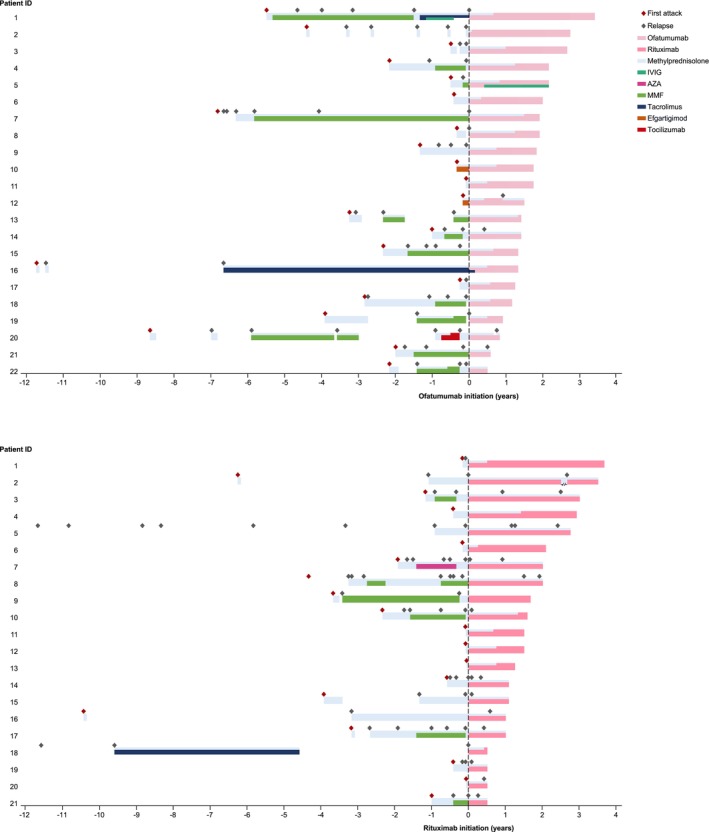
Treatment episodes and relapses before and after initiation of ofatumumab (*n* = 22) and rituximab (*n* = 21) in MOGAD patients. Data prior to treatment are shown up to 12 years before initiation. In the rituximab group, patient 5 experienced the first attack 19.7 years before treatment and had two relapses 13 years prior to treatment. Patient 18 had the first attack 17.6 years before treatment and had relapsed 14.6 years prior to treatment. In Patient 2, the interval between the first and second rituximab treatment episodes was 41 months, this interval has been visually compressed in the figure for clarity and is not shown to scale. AZA, azathioprine; MMF, mycophenolate mofetil; IVIG, intravenous immune globulin; MOGAD, myelin oligodendrocyte glycoprotein antibody–associated disease.

The treatment episodes and relapses of patients treated with ofatumumab and rituximab are shown in Figure 1.

**FIGURE 2:**
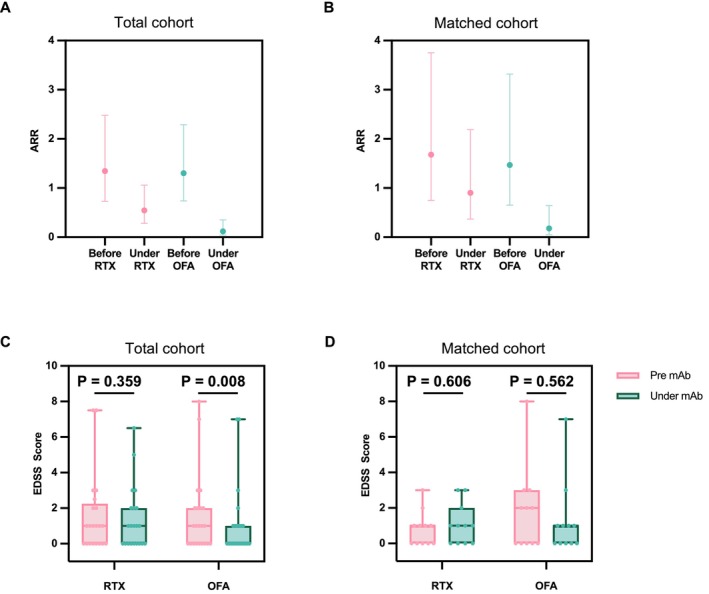
Annualized relapse rates with 95% CI and EDSS scores (median, IQR, and range) before and under ofatumumab (OFA) or rituximab (RTX) treatment in all MOGAD patients and matched cohort (*n* = 11 each). (A) ARR before and under RTX and OFA treatment in the total chort; (B) ARR before and under RTX and OFA treatment in the matched chort; (C) EDSS Scores before and under RTX and OFA treatment in the total chort; (C) EDSS before and under RTX and OFA treatment in the matched chort. IQR, interquartile range; ARR, annualized relapse rate; EDSS, Expanded Disability Status Scale; MOGAD, myelin oligodendrocyte glycoprotein antibody–associated disease; OFA, ofatumumab; RTX, rituximab.

Prior to initiating ofatumumab, patients had a median of 2 relapses (range, 0–6), the ARR was 1.30 (95% CI 0.74–2.29). Over a median treatment duration of 19.5 months (range, 6–41), 4 patients (18.18%) relapsed, and the ARR significantly decreased to 0.12 (95% CI 0.04–0.35) (IRR 10.86, 95% CI 3.22–36.70, *p* < 0.001; Figure 2A).

In comparison, prior to initiating rituximab, patients had a median of 2 relapses (range, 0–10) and the ARR was 1.34 (95% CI 0.73–2.48). Over a median treatment duration of 18 months (range, 6–44), 12 patients (57.14%) relapsed, and the ARR significantly decreased to 0.55 (95% CI 0.28–1.06) (IRR 2.46, 95% CI 1.00–6.06, *p* = 0.050; Figure 2A).

**FIGURE 3:**
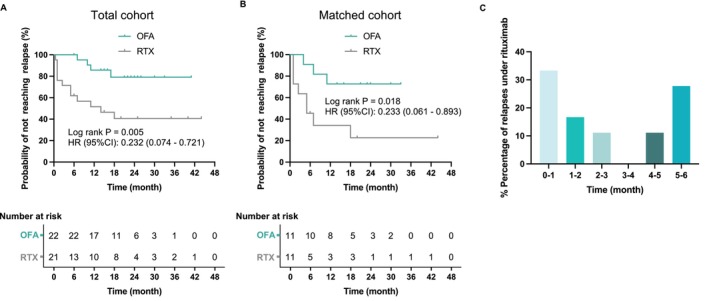
Relapses after initiation of ofatumumab and rituximab in patients with MOGAD. (A, B) Kaplan–Meier curves show a lower risk of relapse in patients treated with ofatumumab compared with those receiving rituximab in both the total cohort (*p* = 0.005) and propensity score–matched cohort (*p* = 0.018). (C) Number of relapses occurring at 0–1, 1–2, 2–3, 4–5, and 5–6 months within a dosing cycle of rituximab. MOGAD, myelin oligodendrocyte glycoprotein antibody–associated disease; OFA, ofatumumab; RTX, rituximab.

Kaplan–Meier analysis was performed to analyze time to first relapse under ofatumumab and rituximab treatment. Data showed that, in the total cohort, patients treated with ofatumumab had a significantly lower risk of reaching relapse (HR 0.232, 95% CI 0.074–0.721, *p* = 0.005; Figure 3A).

Under rituximab, 18 relapses were recorded: 5 (27.8%) occurred within the first month after the initial infusion, 4 (22.2%) between > 1 and ≤ 6 months, 3 (16.7%) between > 6 and ≤ 12 months, and 6 (33.3%) after 12 months. Relapses most commonly occurred shortly after a dose (6/18, 33.3% within the first month) or toward the end of the 6–month dosing interval (5/18, 27.8% between 5 and 6 months). (Figure 3C). In contrast, the four relapses observed under ofatumumab occurred 4, 6, 7 and 11 months after treatment initiation.

After PSM, we further compared the relapse rate between the ofatumumab and rituximab groups (Figure 2B and Table 2). The covariates considered in PSM matching included onset age, gender, disease duration before treatment, number of relapses before treatment, onset phenotype and maintenance therapy before treatment. After 1:1 PSM, most baseline covariates were well balanced, with standardized mean differences below 0.2. Minor residual imbalance was observed for age and baseline relapse count (Table 1). In the ofatumumab group, 72.73% (8/11) of patients remained relapse–free. The median number of relapses during treatment was 0 (range, 0–1), with annualized relapse rate (ARR) of 0.18 (95% CI 0.05–0.64) and a median interval of 7 months (range, 4–11) from treatment initiation to the first relapse. By contrast, in the rituximab group, which had a comparable treatment duration (*p* = 0.088), only 27.28% (3/11) remained relapse–free. The median number of relapses was 1 (range, 0–2), the ARR was 0.90 (95% CI 0.37–2.19), and the median time to first relapse was 3.75 months (range, 1–18). Compared with ofarumumab, rituximab group showed more relapses (*p* = 0.047), and a higher ARR during treatment (IRR 5.10, 95% CI 1.06–24.43, *p* = 0.042, Table 2).

**TABLE 2:** Clinical outcomes between rituximab–treated and ofatumumab–treated patients (*n* = 11 each).

Outcome	Rituximab (*n* = 11)	Ofatumumab (*n* = 11)	*p* value	
Relapse–free patients under treatment, *n* (%)	3 (27.28)	8 (72.73)	0.086	
Relapses under treatment, median (range)	1 (0–2)	0 (0–1)	**0.047**	
ARR before treatment, 95% CI	1.68 (0.75–3.75)	1.47 (0.65–3.32)	—	
ARR under treatment, 95% CI	0.90 (0.37–2.19)	0.18 (0.05–0.64)	—	
EDSS score before treatment, median (range)	1 (0–3)	2 (0–8)	0.385	
EDSS score at the last follow–up, median (range)	1 (0–3)	1 (0–7)	0.748	
Favorable outcome (EDSS score ≤ 2) at the last follow–up, *n* (%)	9 (81.82)	9 (81.82)	1.000	
Time to first relapse after treatment, median (range)	3.75 (1–18)	7 (4–11)	0.279	
Treatment duration^a^, median (range)	12 (6–44)	18 (10–33)	0.088	
Patients who discontinued steroid during treatment, *n* (%)	3 (27.28)	8 (72.73)	0.086	

Outcome	Comparison	IRR	95% CI	*p* value
Relapse rate before treatment	RTX vs. OFA	1.14	0.36–3.60	0.820
Relapse rate after treatment	RTX vs. OFA	5.10	1.06–24.43	**0.042**
Relapse rate before and after ofatumumab	Before vs. After	8.35	1.82–38.40	**0.006**
Relapse rate before and after rituximab	Before vs. After	1.87	0.56–6.21	0.306

*Note:* Data in bold represent *p* values less than 0.05.

Abbreviations: ARR, annualized relapse rate; CI, confidence interval; EDSS, expanded disability status scale; IRR, incidence rate ratio.

^a^ Treatment duration is the time span from the ofatumumab or rituximab initiation to the last follow–up.

Kaplan–Meier analysis demonstrated that, after PSM, patients treated with ofatumumab had a significantly lower risk of a second attack compared with those receiving rituximab (HR 0.233, 95% CI 0.061–0.893, *p* = 0.018; Figure 3B).

Relapse rates were further compared in matched patients with relapsing disease courses (*n* = 8 per group; Figure S1). In this subgroup analysis, 2 of 8 patients on ofatumumab experienced a second attack, whereas 6 of 8 on rituximab relapsed. Kaplan–Meier analysis showed a trend toward a longer time to second attack in the ofatumumab group compared with the rituximab group, although this difference did not reach statistical significance (log–rank *p* = 0.052). However, in this same subgroup (*n* = 8 per group), ARR significantly decreased under ofatumumab treatment (IRR 7.33, 95% CI 1.24–43.45, *p* = 0.028); by contrast, ARR remained comparable before and during rituximab treatment (*p* = 0.583).

Among the four patients who experienced an attack during ofatumumab treatment, three underwent immediate CD19^+^ B–cell testing, revealing B–cell proportions of 0%, 0% and 0.05%, respectively. In contrast, among the twelve patients who relapsed during rituximab treatment, six had immediate CD19^+^ B–cell measurements: three showed 0% B cells, while the others had proportions of 0.17%, 1% and 0.12%. For the remaining patients who relapsed on rituximab, no CD19^+^ B–cell testing was performed, or the tests/results were unavailable.

### 3.3 Factors Associated With Relapse During Treatment

We then entered the type of anti–CD20 mAb, gender, age at onset, age at mAb initiation, prior immunotherapy, concomitant IVIg, initiation time, disease duration before mAb, mAb treatment duration, and the number of pre–treatment relapses into Cox proportional hazards regression to identify factors associated with relapse during therapy with the two anti–CD20 monoclonal antibodies (Table 3). In the adjusted model, ofatumumab—compared with rituximab—was associated with a significantly lower risk of relapse (HR 0.06, 95% CI 0.01–0.26; adjusted *p* = 0.002). Female sex (adjusted *p* = 0.015), initiation of monoclonal antibody therapy after the onset attack (adjusted *p* = 0.035), and longer treatment duration (adjusted *p* = 0.002) were also associated with a reduced risk of relapse. In contrast, a higher number of relapses prior to mAb treatment was associated with an increased risk of relapse (HR 1.79, 95% CI 1.15–2.78, adjusted *p* = 0.026).

**TABLE 3:** Univariate and multivariate Cox proportional hazard analysis of associated factors for relapses during mAb treatment (*n* = 43).

	Univariate analysis		Multivariate analysis	
	HR and 95% CI	Adjusted *p* value	HR and 95% CI	Adjusted *p* value
Anti‐CD20 mAb				
Rituximab	1.00 (Reference)		1.00 (Reference)	
Ofatumumab	0.24 (0.08–0.75)	0.077	0.06 (0.01–0.26)	0.002
Gender				
Male	1.00 (Reference)		1.00 (Reference)	
Female	0.46 (0.17–1.24)	0.226	0.12 (0.03–0.52)	0.015
Age				
At onset	0.98 (0.94–1.02)	0.399	1.32 (0.72–2.41)	0.459
At mAb initiation	0.98 (0.95–1.02)	0.451	0.75 (0.41–1.37)	0.459
Immunotheray before mAb				
Without maintenance therapy	1.00 (Reference)		1.00 (Reference)	
With maintenance therapy	1.59 (0.59–4.29)	0.451	0.20 (0.04–0.94)	0.069
Concomitant IVIg^a^				
Without IVIg	1.00 (Reference)		1.00 (Reference)	
With IVIg	0.00 (0.00—Inf)	0.998	0.00 (0.00—Inf)	0.999
Initiation time				
After a second attack	1.00 (Reference)		1.00 (Reference)	
After onset attack	0.20 (0.03–1.49)	0.226	0.03 (0.00–0.55)	0.035
Disease duration before mAb^b^	1.00 (1.00–1.01)	0.451	1.00 (0.96–1.05)	0.999
Treatment duration of mAb^c^	0.91 (0.85–0.98)	0.077	0.82 (0.74–0.91)	0.002
Number of relapses before treatment	1.17 (0.99–1.40)	0.226	1.79 (1.15–2.78)	0.026

*Note:* Data were used without propensity score matching; data in bold represent *p* values less than 0.05.

Abbreviations: CI, confidence interval; HR, hazard ratio; IVIg, intravenous immunoglobulin; mAb, monoclonal antibody.

^a^ Concomitant IVIg, patients receiving intravenous immunoglobulin in combination with ofatumumab or rituximab.

^b^ Disease duration before mAb is the time span from the onset attack to ofatumumab or rituximab initiation.

^c^ Treatment duration of mAb is the time span from the ofatumumab or rituximab initiation to the last follow–up.

### 3.4 Steroid Tapering

**FIGURE 4:**
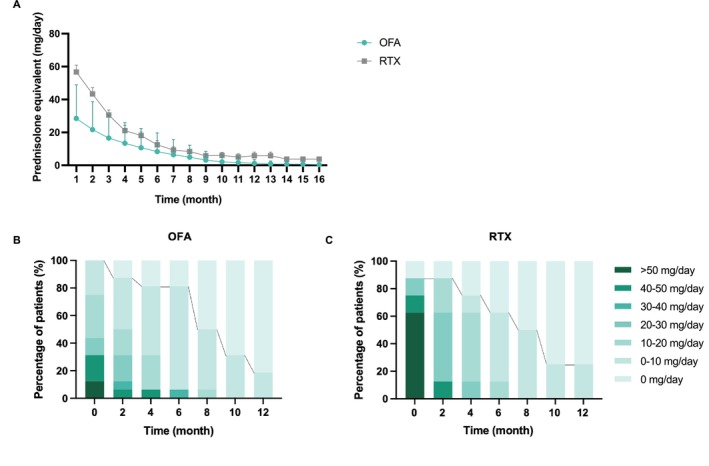
Steroid tapering in relapse–free patients treated with OFA and RTX. (A) Prednisolone–equivalent dose over time in relapse–free patients receiving OFA (*n* = 18) and RTX (*n* = 9). (B, C) Proportion of relapse–free patients receiving concomitant oral corticosteroids at doses of 0, 0–10, 10–20, 20–30, 30–40, 40–50, and > 50 mg/day during 12 months of OFA (*n* = 16) or RTX (*n* = 8) treatment. OFA, ofatumumab; RTX, rituximab.

In both groups, patients who remained relapse–free were able to gradually taper their steroid dose (Figure 4A). Among patients with a treatment duration over 12 months, by the 12–month follow–up, 81.25% (13/16) in the ofatumumab group and 75.00% (6/8) in the rituximab group had discontinued prednisolone (Figure 4B,C). Among all the patients, by the final follow–up, 17/22 patients (77.27%) in the ofatumumab group and 9/21 patients (42.86%) in the rituximab group had discontinued prednisolone and methylprednisolone, respectively.

### 3.5 Disability

In the total cohort of ofatumumab and rituximab, the interval from the most recent attack to treatment initiation was comparable (*p* = 0.111). The EDSS score significantly decreased from a median of 1.0 (range, 0–8.0) prior to treatment to a median of 0 (range, 0–7.0) at the last follow–up in the ofatumumab group (*p* = 0.008; Figure 2C). In contrast, patients treated with rituximab showed no significant change in their EDSS scores, with a median value of 1.0 (range, 0–7.5) at baseline and 1.0 (range, 0–6.5) at the last follow–up (*p* = 0.359; Figure 2C). However, after propensity score matching, EDSS scores before treatment initiation and at the last follow–up were comparable between the two groups (*p* = 0.606 and 0.562; Figure 2D).

### 3.6 Serum MOG– IgG Titer, IgG, IgM and CD19 ^+^ B Cells

In the ofatumumab group, MOG–IgG titers were assessed both before and during treatment. No statistically significant change in MOG–IgG titers was observed (*p* = 0.064; Figure S2A). Both IgG and IgM indices remained stable between baseline and the final follow–up (Figure S2B,C). At the last follow–up, 11 patients treated with ofatumumab demonstrated near–complete depletion of CD19–positive B cells (median 0.07%, range 0.00%–0.40%, Figure S2D).

### 3.7 Retention and AEs

During a treatment period of 19.5 (6–41) months, 2 (9.09%) patients receiving ofatumumab discontinued therapy due to financial burden or disease relapse. Similarly, over a treatment period of 18 (6–44) months, 15 (71.43%) patients discontinued rituximab or switched to alternative therapies. Of these, eleven patients discontinued due to relapses, one patient experienced persistent blood lipid elevation and requested a switch to ofatumumab, and three patients discontinued maintenance treatment following physician recommendations due to stable disease status.

After PSM, A total of 6 patients (54.55%) receiving ofatumumab experienced 8 adverse events (AEs) (Table 4). The most common AE was fever and upper respiratory infection, each reported in 2 of 8 events (25.00%), followed by pruritus, generalized pain, urinary tract infection and blood glucose elevation, each accounting for 1 of 8 events (12.50%). In the rituximab group, 5 out of 11 patients (45.45%) reported a total of 14 AEs. The most frequently observed AEs were upper respiratory tract infection (3/14, 21.43%), followed by pruritus, (2/14, 14.29%), hair loss (2/14, 14.29%) and elevated blood lipid levels (2/14, 14.29%).

**TABLE 4:** Safety profile of ofatumumab (*n* = 11) and rituximab (*n* = 11) in patients with MOGAD.

	OFA (*n* = 11)	RTX (*n* = 11)
Patients with AEs, *n* *(%)	6 (54.55)	5 (45.45)
Number of AEs, *n* † (%)	8	14
Injection–related reaction events	4 (50.00)	2 (14.29)
Fever	2 (25.00)	0 (0.00)
Pruritus	1 (12.50)	2 (14.29)
Generalized pain	1 (12.50)	0 (0.00)
Infection–related adverse events	3 (37.50)	7 (50.00)
Upper respiratory infection	2 (25.00)	3 (21.43)
Vulvitis	0 (0.00)	1 (7.14)
Urinary tract infection	1 (12.50)	1 (7.14)
Pneumonia	0 (0.00)	1 (7.14)
Oral infections	0 (0.00)	1 (7.14)
Blood glucose elevation	1 (12.50)	0 (0.00)
Blood lipid elevation	0 (0.00)	2 (14.29)
Hair loss	0 (0.00)	2 (14.29)
Gastrointestinal complaints	0 (0.00)	1 (7.14)
Leading to discontinuation, *n* *(%)	0 (0.00)	1 (9.09)
Leading to hospitalization, *n* *(%)	0 (0.00)	1 (9.09)
Leading to death, *n* *(%)	0 (0.00)	0 (0.00)

Abbreviations: AEs, adverse events; MOGAD, myelin oligodendrocyte glycoprotein antibody–associated disease, OFA, ofatumumab; RTX, rituximab.

* *n* indicates the number of patients. If a patient had more than one AEs during different treatment episodes, he/she would contribute to every corresponding type of treatment in the analysis.

^†^ *n* indicates the number of AEs.

Ofatumumab demonstrated a higher incidence of injection–related reactions (IRRs) in our cohort compared to rituximab (4/8 vs. 2/14). These IRRs were primarily observed during the first administration of ofatumumab. Among the infusion–related reactions (IRRs) observed in the ofatumumab group, fever was the most common manifestation (2/8), followed by generalized pain (1/8) and rash (1/8). In the rituximab group, IRRs were generally mild, with two patients experiencing transient rash after infusion. Infection–related adverse events occurred in 37.5% of patients receiving ofatumumab and in 50.0% of those receiving rituximab.

One case of a serious infection occurred in a pediatric patient. A school–aged girl began ofatumumab treatment on December 23, 2023, after receiving two courses of high–dose corticosteroid pulse therapy. In January 2024, she developed steroid–induced myopathy and multiple compressive fractures of the thoracic and lumbar vertebrae. Due to prolonged immobilization, she developed a fever and was diagnosed with *Pneumocystis* pneumonia. As a result, ofatumumab treatment was suspended, and she was hospitalized for 1 month, during which she was treated with SMZ for pneumonia. Ofatumumab was resumed in March 2024 after clinical recovery. Thereafter, SMZ was maintained concomitantly with ofatumumab, and no further adverse reactions were observed, allowing therapy to continue without interruption. In the rituximab group, one patient was hospitalized for COVID–19–related pneumonia.

## 4 Discussion

In this study, we evaluated the efficacy and safety of ofatumumab in patients with MOGAD and compared its performance with rituximab. Our findings demonstrated that: (1) ofatumumab significantly reduced the ARR in patients with MOGAD; (2) compared with rituximab, ofatumumab was associated with a lower risk of relapse; (3) by the final follow–up, 17 patients (77.27%) receiving ofatumumab were able to successfully discontinue oral corticosteroids; and (4) ofatumumab exhibited an overall favorable safety profile, although one case of pneumonia was reported, underscoring the need for careful monitoring when administered in combination with high–dose background corticosteroid therapy.

Although rituximab (RTX) is commonly used off–label for treating MOGAD, largely due to its proven success in NMOSD, emerging real–world data highlight its limited efficacy in MOGAD. A growing body of retrospective studies indicates that despite effective B–cell depletion, many MOGAD patients undergoing RTX therapy experience high relapse rates. For example, in the EU Pediatric Demyelinating Disease Consortium study, 102 children who received RTX as a first, second, or third–line treatment continued to relapse despite B–cell depletion [34]. Similarly, an Australian multicenter study involving 33 children and 26 adults with MOG IgG–associated demyelination reported that one of seven patients did not respond to RTX, even though B–cells were fully depleted [35]. In the German Neuromyelitis Optica Study Group (NEMOS), relapses were observed in 6 out of 9 patients receiving RTX [6]. A French prospective study of 16 adult MOG IgG patients found that one–third relapsed despite having less than 0.05% memory B cells [14]. The largest international cohort, reviewing data from 121 patients, showed that while relapse rates decreased by 37%, only 33% of patients were expected to remain relapse–free after two years [15].

This discrepancy in clinical outcomes may reflect the distinct immunopathogenesis of MOGAD, which appears to involve a complex interplay of immune mechanisms. While rituximab effectively targets mature B cells, the pathogenesis of MOGAD may rely not only on these cells but also on other immune components, including T cells, short–lived plasmablasts, and potentially B cells with low CD20 expression that RTX may not efficiently deplete. In addition, a subset of MOGAD patients may exhibit heightened sensitivity to even minimal reemergence of circulating memory B cells, as relapses have been observed when their proportions approach the detection threshold of 0.05% [14]. Due to the high–dose pulse administration of rituximab, an end–of–dose effect may occur, with B cell reappearance observed near the end of 6 month dosing interval. As we observed, a significant proportion of MOGAD relapses in the RTX group occurred during the 5–6 month period following a dose, whereas patients on ofatumumab maintained lower B–cell percentages even at relapse than the rituximab‐treated patients. Collectively, these factors may explain the variable efficacy of RTX in MOGAD and underscore the need for alternative therapeutic strategies to improve clinical outcomes.

In the context of suboptimal efficacy observed with rituximab in treating MOGAD, the use of ofatumumab warrants careful consideration. Our initial experience stems from a single patient with MOGAD who began treatment with this CD20 monoclonal antibody during the COVID–19 pandemic (patient 1 in this study). Due to the convenience of self‐administration and the reduced necessity for hospital visits, the patient opted to initiate treatment with ofatumumab and, notably, did not experience any subsequent disease relapses. This clinical outcome prompted us to reconsider the potential advantages of ofatumumab over rituximab and to speculate on the possible mechanisms underlying its apparent efficacy.

In the current study, ofatumumab was associated with a lower risk of relapse compared to rituximab in treating MOGAD. 81.82% of those treated with ofatumumab remained relapse–free over a median period of 19.5 months, significantly higher than the 42.86% relapse–free rate in the rituximab group. Additionally, ARR was lower in the ofatumumab group. This finding aligns with its successful application in treatment–refractory autoimmune encephalitis, including anti–NMDAR encephalitis [36, 37, 38].

The reasons underlying the differential efficacy of these two anti–CD20 monoclonal antibodies in MOGAD warrant careful consideration. Ofatumumab, approved for the treatment of relapsing multiple sclerosis [39], is a fully human anti–CD20 monoclonal antibody administered subcutaneously, which binds a membrane–proximal epitope of CD20 with high affinity. This distinct binding profile enhances B–cell lysis through both CDC and ADCC, whereas rituximab, which binds a different epitope, induces relatively weaker CDC and relies more heavily on ADCC. As a result, ofatumumab more effectively depletes B cells, even CD20–low B–cell subsets [40, 41, 42]. It may also impact pro–inflammatory Th17 cells [43] which is critical in the pathogenesis of MOGAD. Its low–dose, monthly subcutaneous regimen ensures sustained B–cell depletion, minimizing the possibility of B cell recovery that may occur with rituximab, and more effectively targeting pathogenic B cells residing in the lymph nodes. These combined features support its therapeutic advantages in MOGAD, as suggested by preliminary clinical data as well as the current study [31, 32].

At present, the optimal dosage of rituximab for off–label use in neuroimmunological diseases remains unstandardized. Moreover, establishing an equivalent dose between rituximab and ofatumumab is unlikely. It should be noted that the RTX dosage used in our study is lower than the doses more commonly adopted in Western countries. Nevertheless, our regimen primarily consisting of 500 mg every 6 months (with initial dose of 1000 mg) is widely used in China and has been shown to effectively deplete peripheral B cells and reduce relapses in Chinese patients with AQP4–positive NMOSD [44, 45]. This 500 mg regimen has also been adopted in some MS studies and shown clinical benefits [46, 47, 48]. Importantly, among those MOGAD patients in our RTX group who experienced relapse, the counts of CD19^+^ B cells remained suppressed at the time of relapse, with their proportion not exceeding 1%. Therefore, the superior treatment efficacy of ofatumumab cannot be fully attributed to the relatively lower RTX dose used in our study. However, we recognize that, compared with the conventional two–dose, 2–week induction protocol and higher cumulative dosing regimens commonly used in other settings, the reduced RTX regimen in our cohort may have influenced treatment outcomes to some extent. Therefore, comparisons between OFA and RTX should be interpreted cautiously.

The safety profile of ofatumumab was favorable. Infection–related adverse events occurred less frequently in the ofatumumab group (37.50%, 3/8) compared with the rituximab group (50.00%, 7/14). A serious infection occurred in one pediatric patient, who presented with fever and was diagnosed with Pneumocystis pneumonia, requiring hospitalization 1 month after initiating ofatumumab; however, this event could not be clearly attributed to ofatumumab, as prior exposure to two courses of high–dose corticosteroid therapy may have contributed. The patient resumed treatment after a one–month interruption without further complications. No patients discontinued therapy or died due to treatment–related adverse events. Notably, serum IgG and IgM levels remained stable throughout follow–up. In contrast to rituximab, which has been associated with cumulative immunosuppression and higher rates of hypogammaglobulinemia during prolonged use [42], ofatumumab's low–dose monthly subcutaneous administration appears to impose a lower systemic burden and reduced risk of hypogammaglobulinemia, thereby contributing to improved long–term tolerability [43].

## 5 Conclusion

In conclusion, these findings suggest that ofatumumab may be associated with improved relapse control compared with rituximab in patients with MOGAD, and appears to have a favorable safety profile. However, several limitations should be acknowledged. First, the sample size was modest, which may limit statistical power and the precision of effect estimates. Second, although propensity score matching was performed to mitigate confounding by indication, residual and unmeasured confounding cannot be excluded given the observational nature of the study. Third, rituximab was administered using a reduced–dose regimen in this cohort, which may affect the generalizability of the findings and warrants caution in making direct comparisons between the two therapies. Therefore, larger, prospective studies are warranted to validate these findings, explore biomarkers of treatment response, and define the role of anti–CD20 therapies in the broader landscape of MOGAD management.

## Author Contributions

Yuxin Fan: data curation, formal analysis, investigation, methodology and writing (original draft, review and editing). Lei Zhou, Jingzi ZhangBao, Hongmei Tan and Zhouzhou Wang: writing (review and editing). Chao Quan: conceptualization, formal analysis, investigation; writing – review and editing, funding acquisition, project administration and supervision.

## Funding

This work was funded by the National Natural Science Foundation of China (82171431).

## Ethics Statement

This study was approved by the Medical Ethics Committee of Huashan Hospital. Written informed consent was obtained from each participant.

## Conflicts of Interest

The authors declare no conflicts of interest.

## Supporting information


**Figure S1:** Kaplan–Meier curves of relapses and retention in the ofatumumab and rituximab groups. A lower risk of relapse was observed in patients treated with ofatumumab compared to those with rituximab in the relapsing subgroup * (HR 0.233, 95% CI 0.047–1.164, *p* = 0.052).
* Relapsing MOGAD was defined as the presence of at least clinical attacks.
OFA, ofatumumab; RTX, rituximab.
**Figure S2:** Serum MOG‐IgG titer, IgG, IgM and the percentage of CD19+ B cells in patients treated with ofatumumab. (A) Serum live cell–based MOG‐IgG titers decreased but did not reach statistical significance (*n* = 18, *p* = 0.0637). (B, C) Serum IgG and IgM (*n* = 9 each) levels remained stable before initiation of ofatumumab and at the last follow‐up. (D) The percentage of CD19+ B cells before OFA initiation and at the last follow‐up. MOG‐IgG, myelin oligodendrocyte glycoprotein antibody immunoglobulin G; IgG, Immunoglobulin G; IgM, Immunoglobulin M; OFA, ofatumumab.
**Figure S3:** Distribution of propensity score matching (PSM) probabilities. (A) The entire cohort. (B) The cohort after excluding patients with a monophasic disease course.

## Data Availability

The data that support the findings of this study are available from the corresponding author upon reasonable request.
