# Supplementary material for: Ofatumumab in Myelin Oligodendrocyte Glycoprotein Antibody–Associated Disease: A Comparison With Rituximab
Source: Ann Clin Transl Neurol. 2026 Apr 26:10.1002/acn3.70392. Online ahead of print. doi: 10.1002/acn3.70392 (PMC13394994; doi:10.1002/acn3.70392)
Supplement: Supplementary file 1 — Figure S1: Kaplan–Meier curves of relapses and retention in the ofatumumab and rituximab groups. A lower risk of relapse was observed in patients treated with ofatumumab compared to those with rituximab in the relapsing subgroup * (HR 0.233, 95% CI 0.047–1.164, p = 0.052). * Relapsing MOGAD was defined as the presence of at least clinical attacks. OFA, ofatumumab; RTX, rituximab. Figure S2: Serum MOG‐IgG titer, IgG, IgM and the percentage of CD19+ B cells in patients treated with ofatumumab. (A) Serum live cell–based MOG‐IgG titers decreased but did not reach statistical significance (n = 18, p = 0.0637). (B, C) Serum IgG and IgM (n = 9 each) levels remained stable before initiation of ofatumumab and at the last follow‐up. (D) The percentage of CD19+ B cells before OFA initiation and at the last follow‐up. MOG‐IgG, myelin oligodendrocyte glycoprotein antibody immunoglobulin G; IgG, Immunoglobulin G; IgM, Immunoglobulin M; OFA, ofatumumab. Figure S3: Distribution of propensity score matching (PSM) probabilities. (A) The entire cohort. (B) The cohort after excluding patients with a monophasic disease course. [file ACN3-9999-0-s001.pdf]

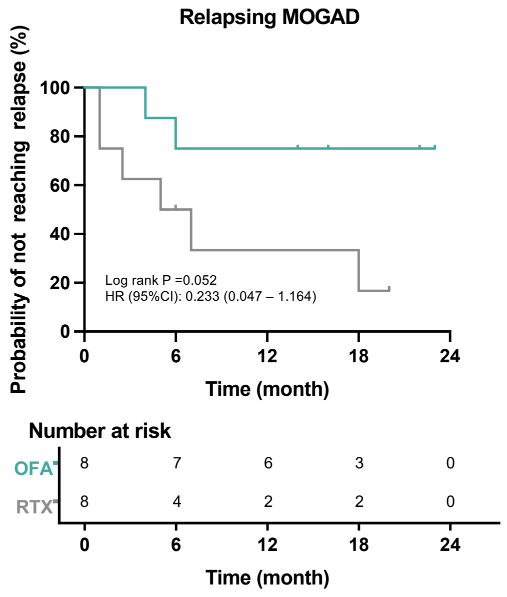

Supplementary Figure 1: Kaplan–Meier curves of relapses and retention in the ofatumumab and rituximab groups.
A lower risk of relapse was observed in patients treated with ofatumumab compared to those with rituximab in the relapsing subgroup * (HR 0.233, 95% CI 0.047 - 1.164, p = 0.052).
* Relapsing MOGAD was defined as the presence of at least clinical attacks.
OFA, ofatumumab; RTX, rituximab.

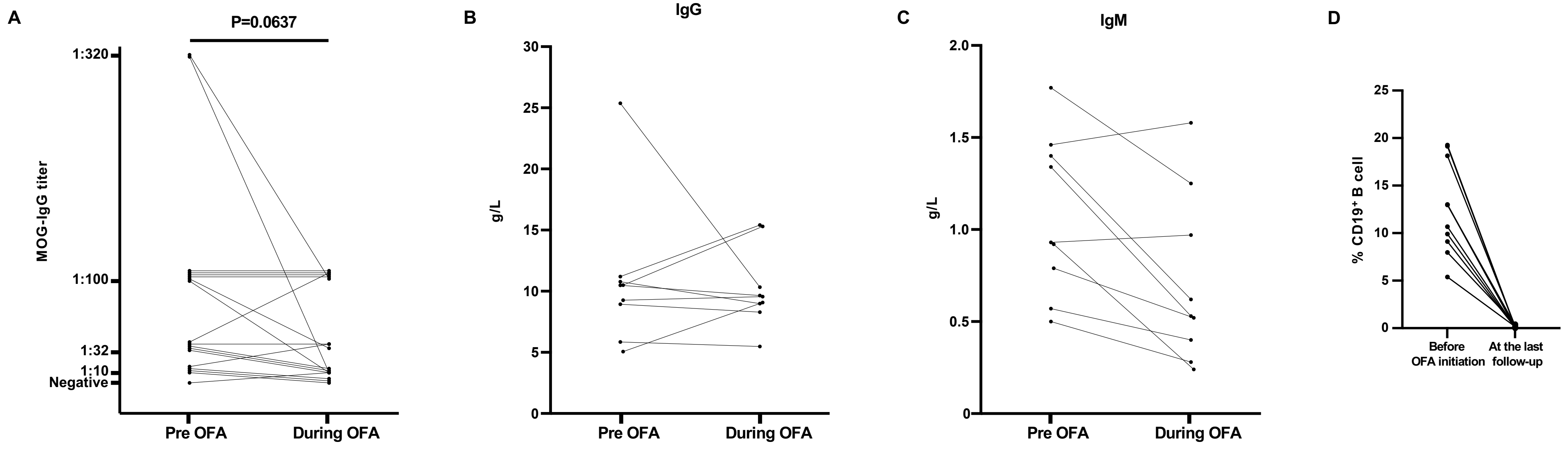

Supplementary Figure 2: Serum MOG-IgG titer, IgG and IgM in patients treated with ofatumumab. (A) Serum live cell–based MOG-IgG titers decreased but did not reach statistical significance (n = 18, p = 0.0637). (B, C) Serum IgG and IgM (n = 9 each) levels remained stable before initiation of ofatumumab and at the last follow-up. (D) The percentage of CD19+ B cells before OFA initiation and at the last follow-up. MOG-IgG, myelin oligodendrocyte glycoprotein antibody immunoglobulin G; IgG, Immunoglobulin G; IgM, Immunoglobulin M; OFA, ofatumumab.

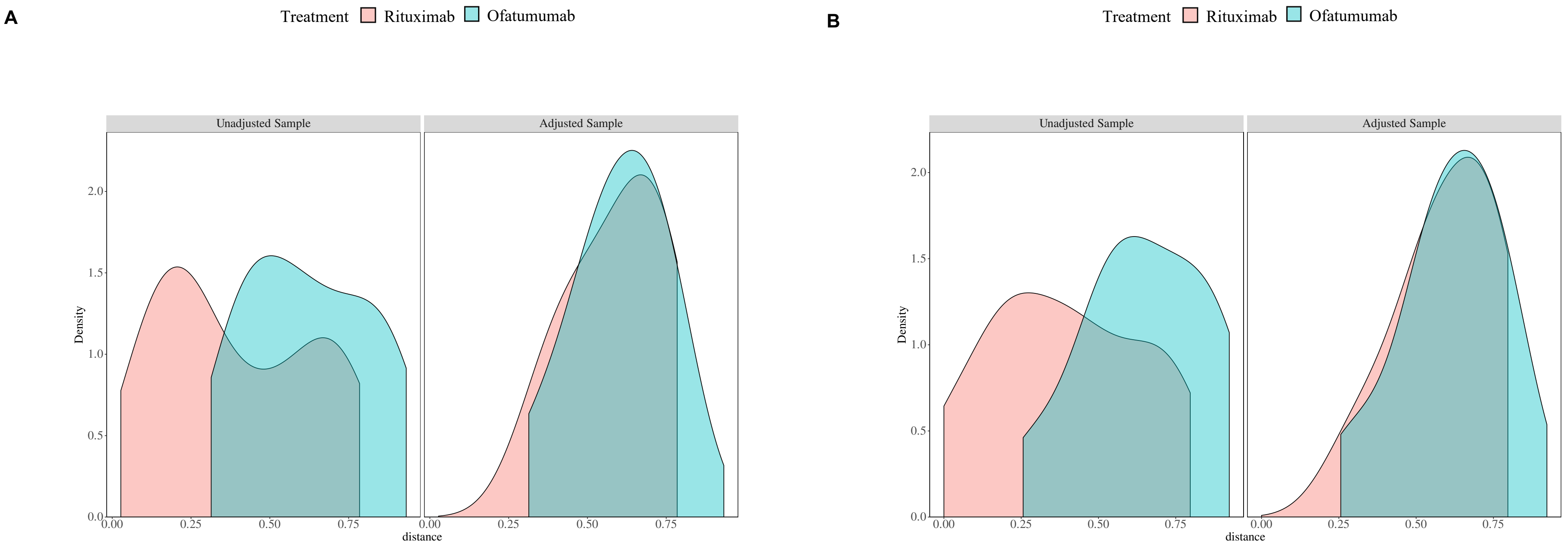

Supplementary Figure 3: Distribution of propensity score matching (PSM) probabilities. (A) The entire cohort. (B) The cohort after excluding patients with a monophasic disease course.
